# N-3 PUFA Prevent Oxidative Stress in a Rat Model of Beta-Amyloid-Induced Toxicity

**DOI:** 10.3390/ph14040339

**Published:** 2021-04-08

**Authors:** Maria Grazia Morgese, Stefania Schiavone, Maria Bove, Anna Laura Colia, Stefania Dimonte, Paolo Tucci, Luigia Trabace

**Affiliations:** Department of Clinical and Experimental Medicine, University of Foggia, 71122 Foggia, Italy; mariagrazia.morgese@unifg.it (M.G.M.); stefania.schiavone@unifg.it (S.S.); maria.bove@unifg.it (M.B.); annalaura.colia@unifg.it (A.L.C.); stefania.dimonte@unifg.it (S.D.); paolo.tucci@unifg.it (P.T.)

**Keywords:** n-3 PUFA, oxidative stress, beta-amyloid, inflammation

## Abstract

Polyunsaturated fatty acids (PUFA) are involved in brain disorders associated to amyloid beta (Aβ) toxicity for which oxidative stress, neurochemical dysfunctions, and neuroinflammation are underlying mechanisms. Here, mechanisms through which lifelong exposure to n-3 PUFA-enriched or n-6/n-3 balanced diets could elicit a protective role in a rat model of Aβ-induced toxicity were investigated. To this aim, we quantified hippocampal reactive oxygen species (ROS) amount, 8-hydroxy-2′-deoxyguanosine and interleukin-10 levels, NADPH oxidase (NOX) 1, NOX2, superoxide dismutase 1, and glutathione contents, as well as plasmatic malondialdehyde. Moreover, in the same experimental groups, we assessed tryptophan, serotonin, and its turnover, kynurenine, and noradrenaline amounts. Results showed increased hippocampal ROS and NOX2 levels, serotonin turnover, kynurenine, and noradrenaline contents in Aβ-treated rats. Both n-6/n-3 balanced and n-3 PUFA enriched diets reduced ROS production, NOX1 and malondialdehyde levels, serotonin turnover, and kynurenine amount in Aβ-injected rats, while increasing NOX2, superoxide dismutase 1, and serotonin contents. No differences in plasmatic coenzyme Q10, reduced glutathione (GSH) and tryptophan levels were detected among different experimental groups, whereas GSH + oxidized glutathione (GSSG) levels were increased in sham animals fed with n-3 PUFA enriched diet and in Aβ-treated rats exposed to both n-6/n-3 balanced and n-3 enriched diets. In addition, Aβ-induced decrease of interleukin-10 levels was prevented by n-6/n-3 PUFA balanced diet. N-3 PUFA enriched diet further increased interleukin-10 and 8-hydroxy-2′-deoxyguanosine levels. In conclusion, our data highlight the possible neuroprotective role of n-3 PUFA in perturbation of oxidative equilibrium induced by Aβ-administration.

## 1. Introduction

Polyunsaturated fatty acids (PUFA), especially the one belonging to the n-3 or n-6 families, are lipid molecules with important biological properties that contribute to the physiological development and functioning of the Central Nervous System (CNS) [1]. For the n-3 family, docosahexaenoic acid (DHA, 22:6n-3) represents around the 40% of PUFA. Eicosapentaenoic acid (EPA, 20:5n-3) is another major component of the n-3 family [2,3,4,5], although its concentration has been reported to be lower in the CNS [6,7]. Long-chain PUFA play a key role in the maintenance of membrane fluidity and in different signaling pathways. This occurs directly or via their bioactive metabolites, including pro-resolvins (n-3 family) [8]. Furthermore, PUFA can modulate several neural signaling crucially involved in the development of neuropsychiatric disorders, such as Alzheimer’s disease (AD) and major depression. These pathologies are highly comorbid and many overlapping alterations in biochemical substrates have been reported [9].

A useful pharmacological tool to investigate this issue is represented by a rodent model of depressive-like phenotype induced by an intracerebroventricular (icv) injection of soluble Aβ_1-42_ in rats. In particular, by using this animal model, we have previously shown that lifelong exposure to n-3 PUFA enrichment in diets (from conception until eight weeks of life) prevented Aβ-induced depressive-like phenotype, in terms of reduced immobility frequency in the forced swimming test, and reverting the decrease in serotonin (5-HT) and neurotrophin levels, observed in the prefrontal cortex of Aβ-treated rats [10].

Recent lines of evidence reported that n-3 PUFA can hamper oxidative stress, i.e., the disequilibrium between the production of reactive oxygen species (ROS) and their degradation, in both peripheral and central body districts [11,12,13]. In turn, ROS amount can be influenced by dietary PUFA [14]. The NADPH oxidase (NOX) enzymes represent one of the major sources of ROS in the CNS. They have been reported to be involved in numerous physiological functions of the brain. In particular, NADPH oxidase 1 (NOX1) and NADPH oxidase 2 (NOX2) enzymes have been also described as key contributors in the development of neuropsychiatric and neurodegenerative diseases [15]. In vitro evidence described NOX2 activation in different cellular subtypes, such as microglia, astrocytes, and neurons, as one of the major underlying mechanisms of Aβ-induced toxicity [16]. Moreover, in vivo observations highlighted beneficial effects of the pharmacological inhibition of NOX enzymes in animal models of Aβ-related pathologies [17]. Soluble Aβ has also been shown to trigger NADPH oxidase-dependent astrogliosis, both in vitro and in vivo [18]. Importantly, it has been demonstrated that PUFA are able to modulate NOX enzymes activity limiting the production of superoxide anion [19]. Among the components of the antioxidant system, superoxide dismutase 1 (SOD1) enzyme, and reduced glutathione (GSH) have been described to play a crucial role in CNS protection against oxidative damage [20,21]. Preclinical and clinical reports have pointed towards a crucial role of n-3 PUFA in preventing dysfunctions of antioxidant pathways observed in neurodegenerative disorders [22,23]. Nonetheless, increased production of reactive species, by inducing an altered intracellular signaling, has also been linked to dysregulation of the inflammatory response.

Neuroinflammation and imbalance between proinflammatory and anti-inflammatory cytokines levels have been related in the development of neuropsychiatric and neurodegenerative disorders [24]. In this regard, PUFA and their derivatives of the n-3 family have been associated with increased production of anti-inflammatory factors, such as interleukin (IL)-10 (for reference, see [25]).

Therefore, in the present study, we investigated the mechanisms through which lifelong exposure to diets enriched in n-3 PUFA or balanced n-6/n-3, with a ratio n-6/n-3 1:1 or 5:1, respectively, could elicit a protective role in a rat model of Aβ-induced toxicity. In particular, a n-3 PUFA enriched diet provided long-life to rats in order to mimic supplementation of these dietary components from gestation to adult life, whereas the n-6/n-3 diet represented a nutritional approach with a more balanced ratio between n-6 and n-3 PUFA. In this model, we quantified ROS and other biomarkers of oxidative stress in the hippocampus of Aβ-treated rats, given the key implication of this brain region in the development of Aβ-induced detrimental effects [26,27]. Moreover, levels of neuroinflammatory biomarkers and associated neurochemical substrates were assessed.

## 2. Results

### 2.1. ROS Production, Levels of 8-Hydroxy-2′-Deoxyguanosine (8-OHdG) and IL-10, Lipid Peroxidation and Coenzyme Q10 (CoQ10)

To evaluate oxidative stress, IL-10, and lipid peroxidation alterations in Aβ-treated animals lifelong exposed to n-3 PUFA enriched or n-6/n-3 PUFA balanced diets, we quantified hippocampal ROS production, 8-OHdG content, IL-10 levels, as well as plasmatic malondialdehyde (MDA) and Co Q10 content.

**Figure 1:**
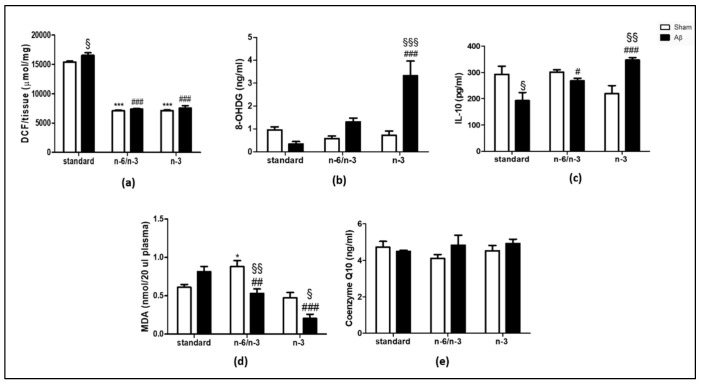
Effects of standard, n-6/n-3 PUFA balanced and n-3 PUFA enriched diets on hippocampal ROS production, 8-OHdG and IL-10 levels, plasmatic MDA, and CoQ10 content in Aβ-treated rats. Measure of hippocampal ROS production (**a**, SHAM standard *n* = 3, Aβ standard *n* = 4, SHAM n-6/n-3 *n* = 4, Aβ n-6/n-3 *n* = 4, SHAM n-3 *n* = 4, Aβ n-3 *n* = 4 ), 8-OHdG (**b**, SHAM standard *n* = 4, Aβ standard *n* = 4, SHAM n-6/n-3 *n* = 4, Aβ n-6/n-3 *n* = 4, SHAM n-3 *n* = 4, Aβ n-3 *n* = 4), IL-10 (**c**, SHAM standard *n* = 4, Aβ standard *n* = 4, SHAM n-6/n-3 *n* = 4, Aβ n-6/n-3 *n* = 4, SHAM n-3 *n* = 3, Aβ n-3 *n* = 4 ), plasmatic MDA levels (**d**, SHAM standard *n* = 4, Aβ standard *n* = 4, SHAM n-6/n-3 *n* = 3, Aβ n-6/n-3 *n* = 4, SHAM n-3 *n* = 5, Aβ n-3 *n* = 4 ) in rats 7 days after Aβ icv injection (Aβ, 4 µM, black bar) or vehicle (SHAM, 5 µL vehicle, white bar) and plasmatic CoQ10 levels (**e**, SHAM standard *n* = 4, Aβ standard *n* = 4, SHAM n-6/n-3 *n* = 4, Aβ n-6/n-3 *n* = 4, SHAM n-3 *n* = 4, Aβ n-3 *n* = 4). Two-way ANOVA followed by Bonferroni multiple comparison test, ^§^ *p* < 0.05, ^§§^ *p* < 0.01, ^§§§^ *p* < 0.001 Aβ-treated rats fed, either, with n-6/n-3 balanced and n-3 PUFA enriched diets versus SHAM rats; * *p* < 0.05, *** *p* < 0.001 SHAM rats fed with n-6/n-3 balanced and n-3 PUFA enriched diets versus SHAM standard diet; ^#^ *p* < 0.05, ^##^ *p* < 0.01, ^###^ *p* < 0.001 Aβ-treated rats fed with n-6/n-3 balanced and n-3 PUFA enriched diets versus Aβ, standard diet. Data were expressed as means ± mean standard error (SEM).

In regard to ROS quantification, in a standard diet receiving rats, Aβ icv injection induced a significant increase compared with sham rats, whereas no differences were detected in n-6/n-3 PUFA balanced or n-3 PUFA enriched diets (Figure 1A, two-way ANOVA followed by Bonferroni multiple comparison test, F_(2,17)_ = 7.016, *p* < 0.05 standard diet group, Aβ vs. sham). N-3 PUFA enriched and n-6/n-3 PUFA balanced diet caused a significant reduction in ROS measurement in sham operated rats versus standard diet fed rats (Figure 1A, two-way ANOVA followed by Bonferroni multiple comparison test, F_(2,17)_ = 576.1, *p* < 0.001 sham n-6/n-3 and n-3 vs. sham standard). Moreover, n-3 PUFA enriched and n-6/n-3 PUFA balanced diet caused a significant reduction in ROS measurement in Aβ-treated rats versus standard diet fed rats (Figure 1A, two-way ANOVA followed by Bonferroni multiple comparison test, F_(2,17)_ = 576.1, *p* < 0.001 Aβ n-6/n-3 and n-3 vs. Aβ standard).

Concerning 8-OHdG levels, results showed higher levels in Aβ-treated animals receiving n-3 PUFA enriched diet, but not standard or n-6/n-3 PUFA balanced, compared with sham (Figure 1B, two-way ANOVA followed by Bonferroni multiple comparison test, F_(2,18)_ = 14.00, *p* < 0.001 Aβ n-3 vs. sham n-3). No differences were detected among sham animals fed with the three different diets, whereas an increase was observed in Aβ-treated rats fed with n-3 PUFA enriched diet compared with standard diet fed animals (Figure 1B, two-way ANOVA followed by Bonferroni multiple comparison test, F_(2,18)_ = 11.99, *p* < 0.001 Aβ n-3 vs. Aβ standard).

Regarding IL-10 expression, we found a reduction in Aβ-treated compared with sham rats in standard diet regimen, while no differences were detected in n-6/n-3 PUFA balanced diet (Figure 1C, Two-way ANOVA followed by Bonferroni multiple comparison test, F_(2,17)_ = 2.429, *p* < 0.05 Aβ standard vs. sham standard). Otherwise, in n-3 PUFA enriched diet, IL-10 levels were significantly increased in Aβ-treated compared with sham rats (Figure 1C, Two-way ANOVA followed by Bonferroni multiple comparison test, F_(2,17)_ = 2.429, *p* < 0.05 Aβ n-3 vs. sham n-3). No significant differences were detected among different diets in sham groups. N-6/n-3 PUFA and n-3 PUFA enriched diet exposure resulted in IL-10 level increase in Aβ-treated animals compared with Aβ-treated rats receiving the standard diet (Figure 1C, two-way ANOVA followed by Bonferroni multiple comparison test, F_(2,17)_ = 13.48, *p* < 0.05 Aβ n-6/n-3 PUFA vs. Aβ standard; *p* < 0.001 Aβ n-3 vs. Aβ standard).

In regard to lipid peroxidation, no differences were detected between Aβ-injected compared with sham rats fed with standard diet, while, in n-6/n-3 PUFA fed animals, Aβ-treated rats reported a decrease in MDA levels compared with sham rats. The same pattern was also found in rats fed with n-3 PUFA enriched diet (Figure 1D, two-way ANOVA followed by Bonferroni multiple comparison test, F_(2,18)_ = 7.443, *p* < 0.01 Aβ-treated n-6/n-3 vs. sham n-6/n-3, *p* < 0.05 Aβ-treated n-3 vs. sham n-3). No significant differences in plasmatic MDA levels in sham rats fed with n-3 PUFA enriched diet compared with sham animals fed with standard diet. Moreover, sham rats receiving n-6/n-3 PUFA balanced diet reported an increase in MDA levels compared with standard diet (Figure 1D, two-way ANOVA followed by Bonferroni multiple comparison test, F_(2,18)_ = 25.06, *p* < 0.05 sham n-6/n-3 vs. sham standard). Ultimately, there was a decrease in plasmatic MDA levels in Aβ-treated rats fed, either, with n-6/n-3 PUFA balanced or n-3 PUFA enriched diets compared with Aβ-treated animals fed with standard diet (Figure 1D, two-way ANOVA followed by Bonferroni multiple comparison test, F_(2,18)_ = 11.56, *p* < 0.01 Aβ-treated n-6/n-3 vs. Aβ standard, *p* < 0.001 Aβ-treated n-3 vs. Aβ standard).

No significant differences were detected in plasmatic CoQ10 among experimental groups (Figure 1E, two-way ANOVA followed by Bonferroni multiple comparison test, F_(2,18)_ = 1.192, n.s.).

### 2.2. NOX1 and NOX2 Expression

In order to evaluate whether lifelong exposure to n-3 PUFA enriched and n-6/n-3 PUFA balanced diets in Aβ-treated rats could induce NOX alterations, we quantified hippocampal NOX1 and NOX2 levels.

**Figure 2:**
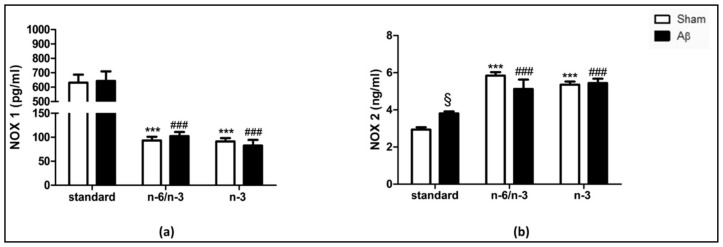
Effects of standard, n-6/n-3 PUFA balanced and n-3 PUFA enriched diets on NADPH oxidases (NOXs) expression in Aβ-treated rats. Effect of standard, n-6/n-3 PUFA balanced and n-3 PUFA enriched diets on hippocampal NOX1 (**a**, SHAM standard *n* = 5, Aβ standard *n* = 8, SHAM n-6/n-3 *n*= 4, Aβ n-6/n-3 *n* = 3, SHAM n-3 *n* = 3, Aβ n-3 *n* = 3) and NOX2 (**b**, SHAM standard *n* = 5, Aβ standard *n* = 8, SHAM n-6/n-3 *n* = 4, Aβ n-6/n-3 *n* = 4, SHAM n-3 *n* = 4, Aβ n-3 *n* = 4) expression in rats 7 days after Aβ icv injection (Aβ, 4 µM, black bar) or vehicle (SHAM, 5 µL, white bar). Two-way ANOVA followed by Bonferroni multiple comparison test, ^§^ *p* < 0.05 Aβ-treated rats fed with standard diet versus SHAM rats; *** *p* < 0.001 SHAM rats fed with n-6/n-3 balanced and n-3 PUFA enriched diets versus SHAM standard diet; ^###^ *p* < 0.001, Aβ-treated rats fed with n-6/n-3 balanced and n-3 PUFA enriched diets versus Aβ standard diet. Data were expressed as means ± mean standard error (SEM).

Our results showed no differences in NOX1 expression between Aβ-injected compared with sham rats fed with standard, n-6/n-3 balanced and n-3 enriched diets (Figure 2A, Two-way ANOVA followed by Bonferroni multiple comparison test, F_(2,20)_ = 0.006, n.s.). NOX1 content was reduced in n-6/n-3 PUFA balanced and n-3 PUFA enriched diets compared with standard diet in sham-operated groups (Figure 2A, two-way ANOVA followed by Bonferroni multiple comparison test, F_(2,20)_ = 59.88, *p* < 0.001 sham n-6/n-3 and sham n-3 vs. sham standard). Moreover, there was a decrease in Aβ-treated rats fed with n-6/n-3 PUFA balanced and n-3 PUFA enriched diets compared with Aβ-treated animals fed with standard diet (Figure 2A, two-way ANOVA followed by Bonferroni multiple comparison test, F_(2,20)_ = 59.88, *p* < 0.001 Aβ n-6/n-3 and Aβ n-3 vs. Aβ standard).

Aβ injection significantly increased NOX2 levels in rats fed with standard diet compared with sham, while no differences were detected in Aβ-treated animals fed with n-6/n-3 balanced and n-3 enriched diets compared with sham rats (Figure 2B, two-way ANOVA followed by Bonferroni multiple comparison test, F_(2,22)_ = 6,304 *p* < 0.05 Aβ standard vs. sham standard). Lifelong exposure to n-3 PUFA enriched or to n-6/n-3 PUFA balanced induced a significant enhancement of NOX2 expression in sham operated rats (Figure 2B, two-way ANOVA followed by Bonferroni multiple comparison test, F_(2,22)_ = 60.09, *p* < 0.001 sham n-6/n-3 and sham n-3 vs. sham standard). Furthermore, n-3 PUFA enriched and n-6/n-3 PUFA balanced diets induced a significant increase of NOX2 expression in Aβ-treated rats (Figure 2B, two-way ANOVA followed by Bonferroni multiple comparison test, F_(2,22)_ = 60.09, *p* < 0.001 Aβ n-6/n-3 and Aβ n-3 vs. Aβ standard).

### 2.3. Effects on Antioxidant Enzymes

To investigate the possible impact of lifelong exposure to n-3 PUFA enriched and n-6/n-3 PUFA balanced diets on the expression of antioxidant enzymes, we quantified SOD1, GSH and GSH+GSSG levels in the hippocampus of Aβ-treated and sham animals.

**Figure 3:**
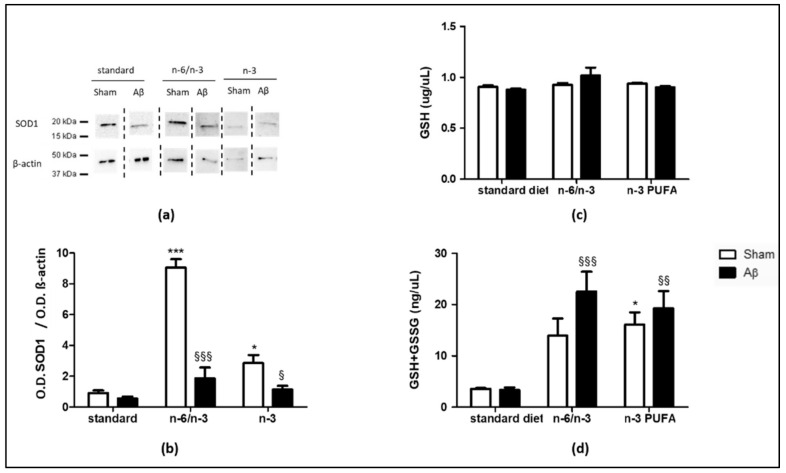
Effects of standard, n-6/n-3 PUFA balanced and n-3 PUFA enriched diets on SOD1, GSH and GSH+GSSG levels in Aβ-treated rats. Representative image of Western blotting (**a**) and quantification of the optical density (**b**, SHAM standard *n* = 3, Aβ standard *n* = 4, SHAM n-6/n-3 *n* = 3, Aβ n-6/n-3 *n* = 4, SHAM n-3 *n* = 4, Aβ n-3 *n* = 4) of SOD1 band normalized to the actin protein value in the hippocampus of rats 7 days after icv injection of Aβ (Aβ, 4 µM, black bar) or vehicle (SHAM, 5 µL, white bar) receiving, from conception until 8-weeks-old, standard, n-6/n-3 balanced or n-3 PUFA enriched diets. GSH (**c**, SHAM standard *n* = 3, Aβ standard *n* = 4, SHAM n-6/n-3 *n* = 4, Aβ n-6/n-3 *n* = 4, SHAM n-3 *n* = 4, Aβ n-3 *n* = 4) levels in rats 7 days after icv injection of Aβ (Aβ, 4µM, black bar) or vehicle (SHAM, 5 µL, white bar) receiving from conception until 8-weeks-old standard, n-6/n-3 balanced or n-3 PUFA enriched diets. GSH + GSSG (**d**, SHAM standard *n* = 3, Aβ standard *n* = 4, SHAM n-6/n-3 *n* = 4, Aβ n-6/n-3 *n* = 4, SHAM n-3 *n* = 4, Aβ n-3 *n* = 4) levels in rats 7 days after icv injection of Aβ (Aβ, 4µM, black bar) or vehicle (SHAM, 5µL, white bar) receiving from conception until 8-weeks-old standard, n-6/n-3 balanced or n-3 PUFA enriched diets. Two-way ANOVA followed by Bonferroni multiple comparison test, ^§^ *p* < 0.05, ^§§^ *p* < 0.01, ^§§§^ *p* < 0.001 Aβ-treated rats fed, either, with n-6/n-3 balanced and n-3 PUFA enriched diets versus SHAM rats * *p* < 0.05, *** *p* < 0.001 SHAM rats fed with n-6/n-3 balanced and n-3 PUFA enriched diets versus SHAM standard diet. Data were expressed as means ± mean standard error (SEM).

Aβ icv injection did not cause differences in SOD1 expression in standard diet exposed animals, while there was a significant reduction in rats either fed with n-6/n-3 PUFA balanced diet or with n-3 PUFA enriched diet compared with sham-operated rats receiving the same diet (Figure 3A,B, two-way ANOVA followed by Bonferroni multiple comparison test, F_(2,16)_ = 40.87, *p* < 0.001 Aβ n-6/n-3 vs. sham n-6/n-3, *p* < 0.05 Aβ n-3 vs. sham n-3). In sham animals, n-6/n-3 PUFA and n-3 PUFA enriched diets significantly augmented SOD1 content compared with standard diet (Figure 3A,B, two-way ANOVA followed by Bonferroni multiple comparison test, F_(2,16)_ = 38.05, *p* < 0.001 sham n-6/n-3 vs. sham standard, *p* < 0.05 sham n-3 vs. sham standard). No significant differences in SOD1 levels were detected among different diets in Aβ groups (Figure 3A,B, two-way ANOVA followed by Bonferroni multiple comparison test, F_(2,16)_ = 16.39, n.s.)

No differences were detected in GSH levels between Aβ-treated and sham groups in all the three diets, as well as among different diets in sham and in Aβ groups (Figure 3C, two-way ANOVA followed by Bonferroni multiple comparison test F_(2,17)_ = 2.061, n.s.). Sham rats fed with n3-PUFA diet showed increased GSH + GSGG levels compared to the same experimental group fed exposed to standard diet (Figure 3D, Two-Way ANOVA followed by Bonferroni multiple comparison test F_(2,17)_ = 16.53, *p* < 0.05 sham n-3 vs. sham standard). Moreover, in Aβ-treated animals, both n-6/n-3 PUFA and n-3 PUFA enriched diets enhanced GSH + GSGG levels with respect to standard diet (Figure 3D, Two-Way ANOVA followed by Bonferroni multiple comparison test F_(2,17)_ = 16.53, *p* < 0.001 Aβ n-6/n-3 vs. Aβ standard, *p* < 0.01 Aβ n-3 vs. Aβ standard).

### 2.4. Effects on Hippocampal Neurochemistry

To corroborate biochemical with neurochemical quantifications, we quantified, in hippocampus, tryptophan (TRP), and serotonin (5-HT) content, 5-HT turnover, kynurenine (KYN), and noradrenaline (NA) levels.

**Figure 4:**
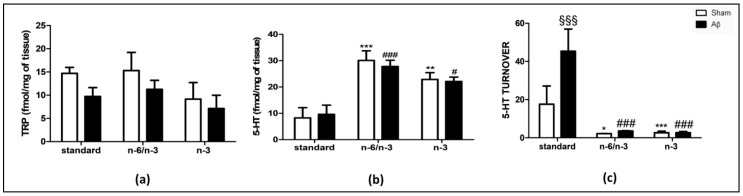
Effects of standard, n-6/n-3 PUFA balanced and n-3 PUFA enriched diets on TRP, 5-HT and 5-HT turnover levels in Aβ-treated rats. Measure of hippocampal TRP (**a**, SHAM standard *n* = 5, Aβ standard *n* = 5, SHAM n-6/n-3 *n* = 5, Aβ n-6/n-3 *n* = 4, SHAM n-3 *n* = 6, Aβ n-3 *n* = 4), 5-HT (**b**, SHAM standard *n* = 4, Aβ standard *n* = 4, >SHAM n-6/n-3 *n* = 6, Aβ n-6/n-3 *n* = 6, SHAM n-3 *n* = 6, Aβ n-3 *n* = 6) and 5-HT turnover (**c,** SHAM standard *n* = 3, Aβ standard *n* = 3, SHAM n-6/n-3 *n* = 5, Aβ n-6/n-3 *n* = 6, SHAM n-3 *n* = 6, Aβ n-3 *n* = 6) contents in rats 7 days after Aβ icv injection (Aβ, 4 µM, black bar) or vehicle (SHAM, 5µL, white bar) receiving, from conception until 8-weeks-old, standard, n-6/n-3 balanced or n-3 PUFA enriched diets. Two-way ANOVA followed by Bonferroni multiple comparison test, ^§§§^ *p* < 0.001 Aβ-treated rats fed, either, with n-6/n-3 balanced and n-3 PUFA enriched diets versus SHAM> rats; * *p* < 0.05, **p < 0.01, *** *p* < 0.001 SHAM rats fed with n-6/n-3 balanced and n-3 PUFA enriched diets versus SHAM standard diet; ^#^ *p* < 0.05, ^###^ *p* < 0.01 Aβ-treated rats fed with n-6/n-3 balanced and n-3 PUFA enriched diets versus Aβ standard diet. Data were expressed as means ± mean standard error (SEM).

As shown in Figure 4A, no differences were detected in TRP content between Aβ-treated and sham groups in all diets, as well as among different diets in sham animals among all groups, and in Aβ-treated animals exposed to n-6/n-3 PUFA and n-3 PUFA diets, compared with Aβ-injected rats fed with standard diet (Figure 4A, Two-way ANOVA followed by Bonferroni multiple comparison test, F_(2,23)_ = 0.14, n.s.).

Concerning hippocampal 5-HT quantification, comparable levels were found between Aβ-treated and sham groups fed with standard diet. Nonetheless, feeding rats with n-3 PUFA enriched and n-6/n-3 PUFA balanced diets for their entire lives resulted in significantly higher 5-HT content in the hippocampal area in sham animals when compared with the same experimental groups receiving standard diet (Figure 4B, two-way ANOVA followed by Bonferroni multiple comparison test, F_(2,26)_ = 21.42, *p* < 0.001 sham n-6/n-3 PUFA vs. sham standard, *p* < 0.01 sham n-3 PUFA vs. sham standard). Furthermore, rats lifelong receiving n-3 PUFA enriched and n-6/n-3 PUFA balanced diets reported significantly higher 5-HT content in the hippocampal area in Aβ animals when compared with the same experimental groups receiving standard diet (Figure 4B, two-way ANOVA followed by Bonferroni multiple comparison test, F_(2,26)_ = 21.42, *p* < 0.001 Aβ n-6/n-3 PUFA vs. Aβ standard, *p* < 0.05 Aβ n-3 PUFA vs. Aβ standard).

Regarding 5-HT turnover, there was a significant increase in Aβ-treated compared with sham rats fed with standard diet, but not in rats receiving n-6/n-3 PUFA or n-3 PUFA enriched diets (Figure 4C, Two-way ANOVA followed by Bonferroni multiple comparison test, F_(2,23)_ = 10.41, *p* < 0.001 Aβ standard vs. sham standard). In addition, a significant decrease in 5-HT turnover was reported in sham treated rats fed with n-6/n-3 PUFA and n-3 PUFA enriched diets compared with sham treated rats fed with standard diet (Figure 4C, two-way ANOVA followed by the Bonferroni multiple comparison test, F_(2,23)_ = 32.80, *p* < 0.05 sham n-6/n-3 vs. sham standard; *p* < 0.001 sham n-3 vs. sham standard). Aβ-treated rats fed with n-6/n-3 PUFA and n-3 PUFA enriched diets showed a significant decrease in 5-HT turnover compared with *Aβ*-treated rats fed with standard diet (Figure 4C, Two-way ANOVA followed by Bonferroni multiple comparison test, F_(2,23)_ = 32.80, *p* < 0.001 Aβ n-6/n-3 vs. Aβ standard; *p* < 0.001 Aβ n-3 vs. Aβ standard).

**Figure 5:**
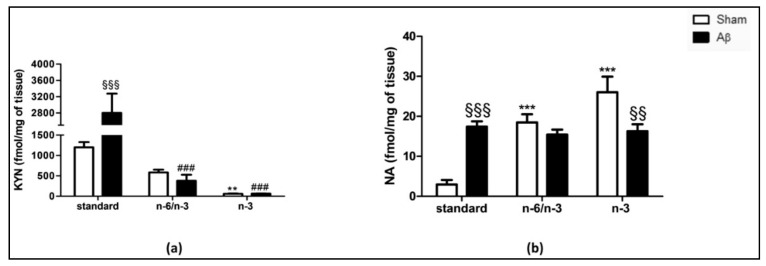
Effects of standard, n-6/n-3 PUFA balanced and n-3 PUFA enriched diets on KYN and NA levels in Aβ-treated rats. Effect of standard, n-6/n-3 PUFA balanced and n-3 PUFA enriched diets on KYN (**a**, SHAM standard *n* = 3, Aβ standard *n* = 4, SHAM n-6/n-3 *n* = 5, Aβ n-6/n-3 *n* = 3, SHAM n-3 *n* = 5, Aβ n-3 *n* = 3) and NA (**b**, SHAM standard *n* = 4, Aβ standard *n* = 4, SHAM n-6/n-3 *n* = 6, Aβ n-6/n-3 *n* = 6, SHAM n-3 *n* = 4, Aβ n-3 *n* = 6) content in the hippocampus of rats 7 days after Aβ icv injection (Aβ, 4 µM, black bar) or vehicle (sham, 5µL, white bar). Two-way ANOVA followed by Bonferroni multiple comparison test, ^§§^ *p* < 0.01, ^§§§^ *p* < 0.001 Aβ-treated rats fed, either, with n-6/n-3 balanced and n-3 PUFA enriched diets versus SHAM rats; ** *p* < 0.01, *** *p* < 0.001 SHAM rats fed with n-6/n-3 balanced and n-3 PUFA enriched diets versus SHAM standard diet; ^###^ *p* < 0.01 Aβ-treated rats fed with n-6/n-3 balanced and n-3 PUFA enriched diets versus Aβ standard diet. Data were expressed as means ± mean standard error (SEM).

The analysis of the TRP metabolite, i.e., KYN, revealed that central Aβ-injection was able to cause a significant increase in the content in the hippocampus of rats fed with a standard diet compared with sham, but not in n-6/n-3 PUFA balanced and n-3 PUFA enriched diets (Figure 5A, two-way ANOVA followed by Bonferroni multiple comparison test, F_(2,17)_ = 6.725, *p* < 0.001 Aβ standard vs. sham standard). Sham animals exposed to n-6/n-3 PUFA and n-3 PUFA enriched diets reported a significant reduction compared with sham standard diet fed animals (Figure 5A, two-way ANOVA followed by Bonferroni multiple comparison test, F_(2,17)_ = 41.49, *p* < 0.01 sham n-3 vs. sham standard). Furthermore, Aβ-treated animals exposed to n-6/n-3 PUFA and n-3 PUFA enriched diets reported a significant reduction in the hippocampal KYN content compared with Aβ-treated standard diet fed animals (Figure 5A, two-way ANOVA followed by Bonferroni multiple comparison test, F_(2,17)_ = 9.72, *p* < 0.001 Aβ n-6/n-3 and Aβ n-3 vs. Aβ standard).

In addition, Aβ-treated rats were characterized by higher hippocampal NA levels compared with sham rats within the standard diet-receiving groups, while no differences were detected in n-6/n-3 PUFA fed animals (Figure 5B, Two-way ANOVA followed by Bonferroni multiple comparison test, F_(2,24)_ = 16.92, *p* < 0.001 Aβ standard vs. sham standard). Conversely, when rats were lifelong exposed to n-3 PUFA enriched diet, Aβ icv injection caused a significant decrease compared with sham (Figure 5B, two-way ANOVA followed by Bonferroni multiple comparison test. F_(2,24)_ = 16.92, *p* < 0.01 Aβ n-3 vs. sham n-3). N-6/n-3 PUFA and n-3 PUFA enriched diets significantly increased hippocampal NA levels in sham groups compared with standard diet (Figure 5B, two-way ANOVA followed by Bonferroni multiple comparison test, F_(2,24)_ = 13.31, *p* < 0.001 sham n-6/n-3 and n-3 vs. sham standard). Lastly, no significant differences NA levels were detected among different diets in Aβ groups (Figure 5B, two-way ANOVA followed by Bonferroni multiple comparison test, F_(2,24)_ = 13.31, n.s.).

## 3. Discussion

The present study showed that enrichment of diet with n-3 PUFA is able to reduce oxidative stress in a rat model of Aβ induced-toxicity.

In our previous experience, we validated a rat model of Aβ-induced depressive-like behavior and such behavioral phenotype was accompanied by reduced cortical 5-HT levels and lower neurotrophin content [28,29]. In addition, our previous findings supported the hypothesis that lifelong exposure to a diet rich in these essential fatty acids of n-3 family could prevent the depressive symptomatology linked to central Aβ injection, with restorage of cortical 5-HT, neurotrophins, and central inflammatory markers [30]. Nonetheless, we have recently shown that depauperation of n-3 PUFA in diet is detrimental for depressive symptomatology in rats and caused accumulation of oligomeric species of Aβ in the hippocampus of rats [31]. Therefore, in trying to understand the mechanisms underlying those effects, and in the tentative to better evaluate a brain area sensitive to Aβ, a peptide whose accumulation in the brain is also associated with AD, we quantified the hippocampal levels of several biomarkers of oxidative stress and inflammation. Indeed, neurons in the hippocampus, especially those in the CA1 region, are populations of neurons very sensitive to the neurodegeneration associated with AD [32,33,34,35]. Consistently, we have previously shown that an icv injection of Aβ can elicit an inflammatory response in the hippocampal area, along with microglia and astrocyte activation [36]. In the present work, we found that Aβ significantly increase hippocampal ROS production in our model. In order to understand whether n-3 PUFA could exert a protective role, animals were lifelong exposed to n-3 PUFA enriched or to n-6/n-3 PUFA balanced diets. In these experimental groups, we found that PUFAs, either in higher ratio or in balanced ratio to n-6 PUFA, significantly reduced ROS productions compared with rats fed with the standard chow diet. In addition, a similar pattern was retrieved for a systemic marker of oxidative stress, such as MDA, obtained as final product of the PUFA peroxidation process [13]. Indeed, N-3 PUFA have been widely described to exert antioxidant and anti-nitrosative effects via the regulation of several proteins implicated in oxidative stress modulation [37]. Furthermore, n-3 PUFA have been reported to decrease microglia activation during chronic inflammatory processes, which are crucially implicated in the development of neurodegenerative diseases [37].

Here, we also demonstrated a specific effect of Aβ icv injection on NOX2 expression in the hippocampus. Indeed, Aβ-treated rats fed with a standard diet showed increased levels of this enzyme with respect to sham animals, whereas no significant difference in NOX1 amount was detected. Our findings are supported by previous evidence showing that NOX2 increase contributed to the development of Aβ-derived neurochemical and behavioral dysfunctions in mice [38]. Moreover, the expression and activation of this enzyme were shown enhanced in microglial cells that mediate amyloid toxicity [39,40,41]. In the same line, increased NOX2 levels in reactive hippocampal astrocytes have been shown to correlate with Aβ neurodetrimental effects [18]. Although in our experimental conditions, we did not detect any significant changes in NOX1 levels between Aβ-treated rats exposed to a standard diet and sham animals, an involvement of this NADPH oxidase isoform cannot be totally excluded. Indeed, a crosstalk between neuronal and microglial NOX1 has been previously described as a possible mediator of Aβ-induced neurodetrimental effects [42]. Thus, quantification of NOX1 in specific cellular subtypes, i.e., neurons or microglia, of Aβ-treated animals might finally result in enhanced levels of this NADPH oxidase isoform. Interestingly, in our experimental conditions, rat exposure to balanced and n-3 enriched diets determined in both sham and Aβ-injected rats a further increase of NOX2 enzyme levels accompanied by a decrease of NOX1 amount in the hippocampus. Taken together, these findings might be interpreted as a possible underlying mechanism finally resulting in the observed ROS decrease in the hippocampus of rats fed with balanced and n-3 enriched diets. Although still speculative, our hypothesis is supported by previous studies showing a similar effect of n-3 fatty acids on NOX2 enzyme in peripheral districts [43] and in other rodent models [44].

In our experimental conditions, we did not detect any difference between sham and Aβ-treated rats in plasmatic levels of CoQ10, an essential electron transporter in the mitochondrial respiratory chain and a potent antioxidant [45]. Supporting our observations, similar systemic levels of this biomarker have been reported in subjects with subclinical and clinical Aβ-induced neurodegeneration compared to healthy controls [46,47]. This finding allows us to hypothesize that other sources of ROS in the CNS, such as mitochondria, may be not primarily involved. However, we cannot totally exclude their possible implication. Indeed, preclinical and clinical evidence have highlighted that Aβ can trigger mitochondrial dysfunctions through numerous pathways, including elevation of ROS production [48,49,50]. Moreover, n-3 PUFA supplementation has been shown to regulate mitochondrial energy metabolism and to prevent mitochondria-derived oxidative stress in the brain following a neurotoxic insult [51]. Besides ROS, other free radicals, such as nitrosative species, were also implicated in the onset and progression of Aβ-associated diseases [52]. In this regard, a suppressive effect of n-3 PUFA against nitrosative stress-induced injury in CNS has been described [37]. Contributing to the observed hippocampal ROS decrease, sham and Aβ-injected rats fed with balanced and n-3 enriched diets showed a significant increase of SOD1 amount in the same brain region. In good agreement with this finding, previous evidence showed elevations of this antioxidant enzyme both in the CNS [53] and in peripheral districts following n-3 PUFA supplementation [54]. In our experimental conditions, no changes were observed in hippocampal GSH levels among experimental groups. In line with this finding, no alterations of GSH amount in this brain region were described in rats exposed to a brain insult and administered with n-3 PUFA [44]. Interestingly, levels of GSH + GSSG in the hippocampus of sham animals was increased by n-3 PUFA enriched diet, whereas, in Aβ-treated animals, both n-6/n-3 PUFA balanced and n-3 PUFA enriched diet were able to enhance GSH + GSSG amount. This result might be interpreted as a further confirmation of the observed ROS reduction induced by PUFA, being GSSG an indicator of the oxidation status [55].

Intriguingly, only in animals exposed to n-3 PUFA enriched diet, Aβ induced a significant increase in 8-OHdG. Although this result seems in apparent contradiction with our previous experience indicating that n-3 PUFA is protective toward Aβ toxicity, in our opinion this data should be interpreted in light of novel literature evidence, considering that 8-OHdG exact physiological role has not been clarified yet. Indeed, it has been proposed that its generation may represent one of the mechanisms through which cells protect themselves against the inflammatory processes induced by oxidative stress [56]. Accordingly, a potent anti-inflammatory effect for 8-OHdG has been reported in vitro by using inflammatory models, such as lipopolysaccharide (LPS)-stimulated microglial cells [57]. Nonetheless, in vivo, it has been evidenced that severe inflammation induced by intraperitoneal LPS injection in mice can be prevented by simultaneous treatment with 8-OHdG, whose effect was reported to be even more powerful than other typical anti-inflammatory agent [58]. Thus, our working hypothesis is that in rats fed with lower n-6/ n-3 ratio, namely n-3 PUFA enriched diet, the pro-oxidant stimulus evoked by Aβ injection can induce the release of 8-OHdG as a protective mechanism ultimately leading to a reduction of inflammatory biomarkers.

In good agreement, n-3 PUFA treated rats receiving Aβ icv injection at hippocampal level showed higher IL-10, an interleukin holding both anti-inflammatory or anti-oxidant properties [59]. Accordingly, it has been reported that recombinant IL-10 was able to block ROS release from mouse peritoneal macrophages [60]. Our results are in good agreement with literature data, considering that it has been shown that the n-6/n-3 ratio was a strong, negative correlate of IL-10, supporting the hypothesis that n-3 fatty acids may exert beneficial effects in patients suffering from pathologies characterized by active inflammation [61], as in our experimental model. Indeed, we found that after the central insult mediated by Aβ icv injection, while the n-6/n-3 balanced diet prevented the reduction in IL-10 caused by the peptide injection, the n-3 enriched diet was able to increase significantly IL-10 levels. The anti-inflammatory effect of n-3 PUFA was also confirmed by the quantification of KYN levels. Indeed, proinflammatory cytokines can stimulate the enzyme indolamine 2,3-dioxygenase (IDO) [62] that converts the precursor of 5-HT, TRP, into KYN. KYN is then transformed in kynurenic acid (KYNA) or quinolinic acid, which may exert neurotoxic effects. The decrease in 5-HT observed in depression has been attributed to the conversion of TRP into KYN instead of 5-HT [63]. Accordingly, in our experimental conditions, lower KYN levels in both PUFA fed groups corresponded to higher 5-HT levels, and according to our previous findings this biochemical event is associated with amelioration of depressive symptomatology [10]. In addition, TRP metabolism in microglial cells can lead to the production of KYN metabolites characterized by reactive oxidative and detrimental properties [64,65]. In this context, Aβ can induce the enhancement of IDO mRNA as well as an increased production of quinolinic acid in human microglia, thus leading to an overstimulation of the KYN pathway [66], as we found in our Aβ-treated rats fed with standard chow diet. In this regard, Aβ-induced microglia activation has been widely reported [67,68] and n-3 PUFA were described to be crucially involved in the modulation of the number of microglial cells, as well as in the regulation of their morphological changes following Aβ icv injection [69]. Thus, the n-3-PUFA beneficial effects observed in our experimental conditions might also represent a protective mechanism against the Aβ-elicited immune response. Indeed, specific molecules with anti-inflammatory roles, such as pro-resolvins, are known to derive from DHA and EPA [70].

Furthermore, in the present experimental condition and in our previous experience [36,71], Aβ injection induces NA increased levels. Based on our previous reports, we know that such increase is a compensatory mechanism associated with the inflammatory stimulus of the peptide. In particular, we have previously demonstrated that icv injection of soluble Aβ causes a significant enhancement of NA levels in the hippocampus already 2 h after its injection and that this effect was prevented by a treatment with an antagonist of IL1-receptors or inhibition of the inducible nitric oxide synthase (iNOS) [71]. Nonetheless, tonic anti-inflammatory properties in the CNS have been attributed to NA [72,73]. Indeed, it can suppress proinflammatory cytokines release and promotes endocytosis and Aβ degradation [67,74,75]. Moreover, NA has been reported to stimulate the release of chemokines with neuroprotective properties in primary astrocytes [76] and to preserve cortical neurons from Aβ-associated toxic effects [77]. In support of NA-related neuroprotection, we previously demonstrated an enhanced 7-days lasting NA amount in the hippocampus in rats treated with soluble Aβ-treated, most likely due to an abnormal neuroinflammatory process, contributing to early neuronal dysfunctions and cognitive alterations [36]. In the present study, PUFAs, and in particular n-3 PUFA enriched diet, was associated with tonically higher NA hippocampal levels, endorsing the hypothesis of a protective anti-inflammatory condition that justify either lower KYN and reduced response to the Aβ insult.

In conclusion, our data indicate the crucial impact of diet and food supplementation in the development of mental disorders. Indeed, our research further endorses the possible neuroprotective role of n-3 PUFA in the onset and progression of neurodegenerative diseases.

## 4. Materials and Methods

### 4.1. Animals

Adult female and male Wistar rats (Envigo, San Pietro al Natisone, Italy) were mated to obtain litters. Animals were constantly exposed to a room temperature of 22 ± 3 °C, relative humidity of 55 ± 5% and to a light/dark cycle of 12 h (light on from 7:00 a.m. to 7:00 p.m.). They can freely access to food and water. We conducted all experimental procedures involving animals in conformity with the institutional guidelines of the Italian Ministry of Health (D.L. 26/2014), the Guide for the Care and Use of Mammals in Neuroscience and Behavioral Research (National Research Council 2004), the Directive 2010/63/EU of the European Parliament and of the Council of 22 September 2010, on the protection of animals used for scientific purposes, as well as the ARRIVE guidelines. Our experimental protocol was approved by the Italian Ministry of Health (protocol number: B2EF8.15-aut.737-2017-PR). During the entire duration of the experimental procedures, we daily monitored animal welfare and we did not detect any signs of animal suffering or distress. Moreover, we made all efforts to minimize the number of animals used in the experiments included in this paper.

### 4.2. Diets

Rats were exposed to the following diets: (a) standard diet; (b) diet containing 6% total fat in the form of only rapeseed oil (n-3, rich in α linolenic acid 18:3n-3); (c) diet containing 6% total fat in the form of 3% of peanut oil plus 3% of rapeseed oil (n-6/n-3, balanced diet) (Laboratorio dottori Piccioni srl, Gessate, Milan Italy). After mating, we randomly assigned dams to one of the above-mentioned diets and the attributed diet was provided during both the gestation and lactation periods. The same diet was also continued in the offspring after weaning and for the next five weeks. Hence, all experimental procedures were performed in eight week-old male rats.

### 4.3. Aβ Administration

The human Aβ1–42 peptide (sequence: Asp-Ala-Glu-Phe-Arg-His-Asp-Ser-Gly-Tyr-Glu-Val-His-His-Gln-Lys-Leu-Val-Phe-Phe-Ala-Glu-Asp-Val-Gly-Ser-Asn-Lys-Gly-Ala-Ile-Ile-Gly-Leu-Met-Val-Gly-Gly-Val-Val-Ile-Ala) was purchased from Tocris (Bristol, UK) and was freshly prepared, as previously describe (vehicle: sterile double distilled water; final concentration 4 μM) [28]. Seven week-old rats were anesthetized by injecting intraperitoneally (i.p.) a solution (0.85 mL/kg) containing the following drugs: ketamine (100 mg/mL), xylazine (100 mg/mL) and acepromazine (10 mg/mL), dissolved in saline. Rats were then secured in a stereotaxic frame (David Kopf Instruments, Tujunga, CA, USA). Icv infusion was performed based on the following coordinates from bregma according to the atlas of Paxinos and Watson [78]: AP = −0.5, ML = +1.2 and DV = −3.2. The incisor bar was set at -3.3 mm. Five μL of soluble Aβ were delivered using a 25 μL Hamilton micro-syringe, and the infusion rate was set at 2 μL/min over 2.5 min. To avoid elapsing during the removal of the needle used for the infusion, it was further left in place (5 min). Control animals (SHAM) were infused with vehicle only, since our previous unpublished observations highlighted that reverse Aβ42-1 was not distinguishable from vehicle alone. The correct placement of the needle was verified when dissecting brain. Experimental procedures were conducted in both SHAM and Aβ-treated rats 7 days after icv administration.

### 4.4. Post-Mortem Tissue Analysis

Rats were euthanized and, immediately after, plasma was collected, and brains were removed. They were then cooled on ice for hippocampus dissection, according to the atlas of Paxinos and Watson [78]. Collected hippocampi were frozen and stored at −80 °C, until the carrying out of the analyses.

### 4.5. High-Performance Liquid Chromatography (HPLC) Quantifications

Serotonin (5-HT), noradrenaline (NA), triptophan (TRP), kynurenin (KYN), 5-hydroxyindolacetic acid (5-HIAA) hippocampal concentrations were determined by high performance liquid chromatography (HPLC) coupled with an electrochemical detector (Ultimate ECD, Dionex Scientific, Milan, Italy). Separation was accomplished by a LC18 reverse phase column (Kinetex, 150 mm × 3 mm, ODS 5 μm; Phenomenex, Castel Maggiore-Bologna, Italy). Detection was performed through a thin-layer amperometric cell (Dionex, ThermoScientifics, Milan, Italy) with a 5 mm diameter glassy carbon electrode. For NA and 5-HT detection, the working potential was set at 0.400 V (vs Pd) [79]. For TRP and KYN detection, the working potential was set at 750 V (vs Pd) [31]. A solution of 75 mM NaH2PO4, 1.7 mM octane sulfonic acid, 0.3 mM EDTA, acetonitrile 10%, in distilled water, buffered at pH 3.0 was used as mobile phase. All chemicals and reagents for HPLC experiments were purchased from Sigma Aldrich, Milan Italy. An isocratic pump (Shimadzu LC-10 AD, Kyoto, Japan) was used, with a flow rate of 0.650 mL/min. Chromeleon software (version 6.80, Thermo Scientific Dionex, San Donato Milanese, Italy) was used for acquisition and integration of data. Results were expressed as fmol/mg of tissue. The following formula: 5HIAA/5-HT was considered to calculate the 5-HT turnover in the hippocampus.

### 4.6. Enzyme-Linked Immunosorbent Assays (ELISA)

Ten volumes of PBS with protease inhibitors were used to homogenize samples, as previously described [80]. Commercially available ELISA kits were used to assess hippocampal levels of 8-OHdG (JaICA, Shizuoka, Japan), NOX1 (MyBiosource, San Diego, CA, USA), NOX2 (MyBiosource, San Diego, CA, USA), IL-10 (MyBiosource, San Diego, CA, USA), according to the manufacturer’s instructions. CoQ10 levels were assessed in plasma (MyBiosource, San Diego, CA, USA). Duplicates were analyzed for both standards and samples to avoid intra-assay variations.

### 4.7. GSH + GSSG/GSH Assay

GSH + GSSG levels and GSH amount were determined by the GSH + GSSG/GSH Assay, using a commercially available kit (Abcam, Cambridge, UK), following manufacturer’s instructions.

### 4.8. Western Blotting

Pierce BCA Assay (Thermo Fisher Scientific, Cleveland, OH, USA) was used to quantify protein content of the hippocampal homogenate. SDS-PAGE precast gels (Bio-Rad Laboratories Inc., Segrate, Italy) were used to separate thirty micrograms of proteins that, after, were transferred onto nitrocellulose membranes (Bio-Rad Laboratories Inc., Segrate, Italy). Rabbit monoclonal anti-SOD1 antibody (1:1000, Abcam, Cambridge, UK) was used. After HRP-conjugated, anti-rabbit secondary antibody (1:25,000, Abcam, Cambridge, UK) was used and the immune complex was detected by chemiluminescence captured on the ChemiDoc MP system (Bio-Rad Laboratories Inc., Segrate, Italy). Optical band density was quantified using ImageJ software (http://rsb.info.nih.gov/ij/, accessed on 7 January 2021) and normalized with β-actin (1:5000, Abcam, Cambridge, UK).

### 4.9. ROS Measurement

Hippocampal ROS amount measurement was conducted as previously described [81,82], by using the fluorogenic dye 2′-7′dichlorofluorescein diacetate (Sigma Aldrich, Milano, Italy) [83]. Tissue homogenization was performed in PBS 1X (pH = 7.4). The fluorogenic dye (final concentration of 5 mM) was then added to homogenized samples. After an incubation at 37 °C for 15 min, samples were centrifuged (10 min, 4 °C, 12,500 rpm). Five ml PBS 1X were used to resuspend the pellet that, after, was put on ice for a period of 10 min. Samples were incubated again at 37 °C for 1 h and then analyzed by a fluorometer (Filter Max F5, Multi-Mode Microplate Reader, excitation length 475 nm, emission length 535 nm). Results were expressed as μmol DCF/mg of tissue.

### 4.10. MDA Assay Measurement

Plasma samples were used to perform MDA assay by a commercially available kit (Sigma-Aldrich, Milano, Italy), as previously described [81,84], according to the manufacturer’s instructions. Each sample and standard analysis was performed in duplicate to avoid intra assay variations. Duplicates were analyzed for both standards and samples to avoid intra-assay variations. Results were expressed as nmol/20 μL plasma.

### 4.11. Statistical Analysis

Results from all experiments were expressed as means ± mean standard error (SEM). GraphPad 5.0 software for Windows (GraphPad Software, San Diego, CA, USA) was used to perform statistical analysis. Bartlett’s test was conducted to verify data normality. Data were then analyzed by Two Way ANOVA, followed by Bonferroni multiple comparison test. For all tests, P value was set at 0.05.

## Data Availability

The data reported in this study are available in this manuscript.
